# A hierarchical approach to combine histological grade and immunohistochemical factors to identify high-risk luminal breast cancers

**DOI:** 10.3332/ecancer.2022.1382

**Published:** 2022-05-04

**Authors:** Felipe Andrés Cordero da Luz, Eduarda da Costa Marinho, Camila Piqui Nascimento, Lara de Andrade Marques, Patrícia Ferreira Ribeiro Delfino, Rafael Mathias Antonioli, Rogério Agenor de Araújo, Marcelo José Barbosa Silva

**Affiliations:** 1 Center for Cancer Prevention and Research, Uberlandia Cancer Hospital, Av Amazonas nº 1996, Umuarama, Uberlândia, Minas Gerais, MG 38405-302, Brazil; 2 Laboratory of Tumor Biomarkers and Osteoimmunology, Institute of Biomedical Sciences, Federal University of Uberlandia, Av Pará nº 1720, Bloco 6T, room 07, Umuarama, Uberlândia, Minas Gerais, MG 38405-320, Brazil; 3 Medical Faculty, Federal University of Uberlandia, Av Pará nº 1720, Bloco 2U, Umuarama, Uberlândia, Minas Gerais, MG 38400-902, Brazil; a https://orcid.org/0000-0002-9381-4913; b https://orcid.org/0000-0002-1307-9104; c https://orcid.org/0000-0002-0955-8559; d https://orcid.org/0000-0002-2734-8352; e https://orcid.org/0000-0002-2196-9318; f https://orcid.org/0000-0003-3886-1562; g https://orcid.org/0000-0003-4653-6786; h https://orcid.org/0000-0002-5807-4286

**Keywords:** breast neoplasms, oestrogen receptor alpha, Ki-67 antigen, neoplasm grading, progesterone receptors

## Abstract

**Background:**

The luminal subtype accounts for ~70% of newly diagnosed breast cancer patients. Although it has a better prognosis, approximately 30% of them develop a late relapse. Identifying those patients is of interest to improve treatment decisions.

**Methods:**

A retrospective observational, single-centre study based on data from medical records of 572 non-metastatic (I–III) invasive ductal breast carcinoma patients, 448 with luminal tumours and 124 with triple-negative tumours. Kaplan–Meier, Cox regression and time-dependent Cox regression were carried out to obtain the prognosis value of risk factors.

**Results:**

During a median observation of 5.5 years, 105 distant metastasis events and 105 all-cause deaths were observed. In addition to known clinicopathological factors (i.e., age, tumour size and lymph node metastasis), the high semi-quantitative expression of both hormone receptors was associated with distant metastasis-free survival (DMFS) (adjusted hazard ratio (HaR): 0.524 (0.316–0.867), *p* = 0.012) and overall survival (OS) (adjusted HaR: 0.486 (0.286–0.827), *p* = 0.008). The stratified analysis made it possible to identify risk modification factors. Subsequent stratification by histological grade, Ki-67 and semi-quantitative PR expression or, mainly, the composite semi-quantitative expression of hormone receptors (cHR) enabled the identification of luminal breast cancer patients of adjuvant schema at higher risk for metastasis and death. However, initial analyses including patients of neoadjuvant therapy pointed to a path of subsequent stratification by cHR and histological grade, also enabling grouping of luminal breast cancer patients with similar prognosis for DMFS (cHR ≤ 4+ G2 or G3 versus triple-negative, adjusted HaR: 0.703 (0.415–1.189), *p* = 0.189) and OS (cHR ≤4+ G2 or G3 versus triple-negative, adjusted HaR: 0.662 (0.403–1.088), *p* = 0.104).

**Conclusion:**

The semi-quantitative expression of both cHR, Ki-67 proliferation index and histological grade can identify luminal breast cancer patients at greater risk of developing metastasis and death when combined in a hierarchical fashion, and could be useful for a better prognosis stratification in services from low- and middle-income countries.

## Background

Worldwide, breast cancer is responsible for the majority of cancer deaths in women [1], mainly in the recurrence of the development of distant metastasis [2]. However, breast cancer is a highly heterogeneous disease, being classified as luminal (oestrogen receptor (ER)/ progesterone receptor (PR)-positive, HER2-negative), HER2-enriched (HER2-positive) or triple-negative (negative to ER, PR and HER2) [3].

About 70% of the patients are of the luminal subtype [3, 4]. Although the luminal subtype has a better prognosis than HER2-enriched and triple-negative cancers [5, 6], 25%–30% of them develop resistance to standard endocrine therapy and develop distant relapse in a late pattern [7, 8]. Thus, only the expression of hormone receptors is not sufficient for identifying patients at higher risk of relapse.

Prognostic improvements were further obtained by the use of the proliferation index by Ki-67 [9], the expression level of the progesterone receptor [10] and the histological grade classification by the Nottingham Grading System [11, 12]. However, the current gold standard for identifying luminal patients (ER/PR-positive, HER2-negative) at high risk of developing distant recurrences are genomic tests such as MammaPrint [13–18], Prosigna (Pam50) [17–20], EndoPredict [17, 18, 21, 22] and, especially, Oncotype Dx [17, 18, 23–27]. Despite having positive cost-effectiveness when considering the benefit in quality of life [28–31], the high cost of these tests makes their access unfeasible for a great majority of patients, mainly in low- and middle-income countries [30, 31], hence the importance of using more accessible risk ratings. Nonetheless, the usually evaluated factors in the pathological routine for the diagnosis of patients with breast cancer have a good correlation with such genomic tests [32–37]. But it is necessary to assess these factors together to classify as low- or high-risk diseases.

There are currently two consolidated important classification systems based on histopathological and molecular (immunohistochemistry) factors, but with different approaches. The system by the American Joint Committee on Cancer (AJCC) aims at staging classification by grouping patients with similar overall survival [25], while the St Gallen system aims to group patients with similar risks of developing distant relapses [17, 38]. Even though incorporating almost the same histopathological and immunohistochemical factors in both systems, there are certain discrepancies in the way they are incorporated, such as the qualitative expression of hormone receptors in AJCC [25] and semi-quantitative expression of PR in St Gallen [17, 38]. Possible explanations are the effect of these factors on the analysed outcome or the way to classify them. Another is the interaction of these factors with each other, in which both systems cannot integrate more solidly [17, 25].

This retrospective study aims to assess whether immunohistochemical markers and degree of differentiation can be hierarchically integrated to identify patients with luminal breast cancer at high risk of developing distant metastasis and whether this classification also has a prognosis value for overall survival.

## Methods

### Study design

A retrospective observational study analysing data collected from the medical records of patients with breast cancer treated at the oncology sector of the Federal University of Uberlandia between January 1999 and December 2019.

### Ethical aspects

This study was approved by the Human Research Ethics Committee (protocol number 803.826/14) of the local Institution and followed all the ethical principles of the Declaration of Helsinki and its subsequent amendments or comparable ethical standards. The informed consent form was waived according to the type of study carried out.

### Classifications and outcomes

Based on the anatomopathological examination results, and not on the medical notes, all patients were reclassified in their pathological TNM stage according to the Seventh Edition of the AJCC [25]. The highest classification of tumour size (*T*) and lymph node metastasis (*N*) between clinical and pathological staging was selected for prognosis factor analysis.

Tumours were classified as luminal when there was a lack of HER2 superexpression/amplification (0/1+ by immunohistochemistry (IHC) and/or lack of *ERBB2* amplification by *in situ* hybridisation technique), and both hormone receptors (ER and/or PR) were expressed in less than 1% of tumour cells. Tumours were classified as triple-negative whenever there was a lack of HER2 superexpression/amplification (0/1+ by IHC and/or lack of *ERBB2* amplification by *in situ* hybridisation technique), and less than 1% of tumour cells expressed both hormone receptors (ER and/or PR) [4].

Patients’ ages were classified by two cut-off values according to the analysed outcome: ≥70 for overall survival due to shorter life expectancy and higher risk of all-cause death receiving fewer treatments [39, 40], and ≤40 for distant metastasis due to increased risk of relapses in younger patients, especially in luminal breast cancer patients [41].

The systemic and radiotherapy treatments were considered adequate whenever patients received treatment as indicated by the current guidelines [10, 17, 42, 43]. Patients were classified as having received or not having received treatment if there was correct adherence or not to the treatment protocols, respectively, as in a previous study [44].

Survival analysis considered the time from diagnosis to censoring or development of the event of interest. As primary and secondary outcomes, the development of distant metastases and general deaths, respectively, were analysed.

### Histological and immunohistochemistry methods

Histological grade was evaluated by haematoxylin and eosin stain of histological slides and classification was carried out in accordance with the Nottingham system [12, 45].

Immunohistochemical data were retrieved from IHC reports. IHCs were prospectively carried out at the local laboratory of the institution, according to the good practice guidelines preserved through time [4, 46]. Detection and revealing were carried out by an avidin–biotin–peroxidase system.

According to standard guidelines of the pathology laboratory at the cited institution, immunological and histological analyses were carried out by one pathologist and independently confirmed by another pathologist.

### Semi-quantitative expression classification of hormone receptors

The majority of the patients had their hormone receptor (HR) expression reported in the cross-system by Sannino and Shousha [47]. This is an ordinal classification system for hormone receptor expression based on the percentage of tumour cells expressing the marker and the intensity of the label. For standardisation, the few reports in intensity and/or percentage of expressing tumour cells were transformed to this cross system, as available in the literature [46, 47].

Tumours were classified according hormonal receptor positivity as 1+ whenever ≥1% and ≤10% of tumours cells expressed the receptor with low intensity; 3+ whenever 60%–90% of tumours cells expressed the receptor with moderate/strong intensity; and 4+ whenever >90% of the tumour cells expressed the receptor with strong intensity. Tumours with an expression greater than 1+ and less than 3+ are considered undefined as it is a very broad spectrum [46, 47]. Therefore, these patients were not included in the initial analyses.

Posteriorly, patients were also reclassified by the sum of both HR expression patterns in a system from 1 to 8 points. As no consensus has been established for 2+ classification, patients in this situation were included only if the other HR had an expression pattern of 4+, being classified as 5, or 1+, being classified as 3, or 0, being classified as 2. The 2+ classification was only considered if it was accompanied by the percentage and labelling of both receptors to enable the sum of their semi-quantitative expressions. The expression of both hormone receptors, and their composite (sum), was evaluated as continuous variables.

### Eligibility criteria

Female patients with invasive ductal carcinoma of no special type histology were included. Patients were excluded by the following criteria: lobular histology or presence of special components (*n* = 713); exclusive *in situ* disease (*n* = 104); synchronic metastatic disease (considering diagnosis within the first 6 months) (*n* = 174); missing histopathological data (histological grade), lysed/destroyed tumour, incomplete immunohistochemistry (absence of ER, RP, HER2, or Ki67) and/or indeterminate HER2 (2+ with indeterminate/without hybridisation method) (*n* = 411); failure to perform surgery (*n* = 6); neoadjuvant radiotherapy (*n* = 6); bilateral cancer (*n* = 2); more than one primary cancer (*n* = 4); follow-up time less than 180 days (6 months) from diagnosis to death/censoring to avoid non-diagnosed synchronous metastasis (*n* = 19); and HER2 overexpression/amplification (*n* = 128).

From a total of 2,186 records, 1,567 were excluded based on the aforementioned criteria. After exclusion criteria were applied, 619 patients with non-metastatic, invasive ductal carcinoma of no special type histology of luminal or triple-negative subtype with complete clinicopathological reports were included in the study.

### Statistical analysis

Distributions were analysed using the Kolmogorov–Smirnov test. Continuous variables with normal distribution were described as mean (± standard deviation) and non-parametric variables as median (minimum–maximum); categorical variables were described as frequencies.

The association between interdependent categorical factors was evaluated using Pearson’s *χ*^2^ test. The association was considered positive (direct) when the adjusted standardised residuals had a value >(+2.0) and considered negative (indirect/inverse) when the value was <(–2.0).

To assess the degree of agreement in the classification of patients between two different approaches, Cohen’s Kappa test was used.

To assess the bivariate correlation in the presence of an ordinal variable, Spearman’s correlation test was used.

To determine the independent prognosis value of the variables, the Cox proportional hazards regression model was used. The proportionality of categorical variables was assessed using the Kaplan–Meier (KM) estimator curve associated with the Log-Rank test.

The proportionality, and linearity, of the risk of continuous variables, was tested by categorising them into defined percentiles. Risk proportionality was assumed as long as and when there were constant risks, evaluated by the KM estimating curve associated with the Log-Rank test. Associated with this, the correlation between the partial residuals generated by the univariable Cox regression with the observation time for each outcome was evaluated; time dependence was assumed in the presence of correlation between these variables and visually analysed by a scatterplot.

In violating the prerequisite of proportionality of risks, time-dependent Cox regression models were used. For categorical variables, observation times were categorised as the time of inflection (crossing) between the curves, and the T_COV variable with interaction (*) with this categorisation was used. Univariable analysis of prognosis factors in a time-dependent context was carried out in the presence of the T_COV* variable.

The Cox regression model with the maximum prognosis value for the analysed cohort was obtained using a two-block analysis. In the first block, candidate factors were included and the Stepwise Forward Wald method was used with an entry *p*-value equal to 0.10 and output *p*-value equal to 0.05 to reduce covariate collapsibility [48] and overfitted models; in the second block, the forced entry method of known prognosis factors, like *T*, *N* and age, and treatments was used, when pertinent. A second model was built inserting all variables with a *p*-value < 0.05 in univariable analysis, or with a known theoretical value, to correct any possible overfitting. In risk stratification analyses, the models were always adjusted by *T* and *N* for distant metastasis-free survival (DMFS), and *T*, *N* and age for overall survival (OS). The simple contrast was used to establish the reference level for categorical variables; the repeated contrast was used to carry out clustering of levels with similar prognosis within a variable.

The aforementioned statistical analyses were carried out with IBM SPSS v25.0.

Using Jamovi v1.6.5.0 software, continuous variables were categorised according to optimal cut-off points obtained by the system after survival analysis of continuous predictors.

A *p*-value < 0.05 was considered significant for all the aforementioned analyses.

## Results

### Analysis of risk factors in patients with luminal breast cancer

Of the 619 patients included in the study, 495 and 124 were identified as having luminal and triple-negative breast cancer, respectively. In a median observation period of 64.9 months, 112 events of distant metastasis and 112 deaths were observed. Detailed data are described in Table 1.

To identify the risk factors associated with the development of distant metastases and death in patients with luminal breast cancer, analyses only in this subgroup were carried out.

Survival analyses for continuous variables were carried out for Ki-67 and the quantitative expression of the hormone receptors (HRs) and the optimal cut-off points were identified. For Ki-67, the survival analysis showed no association with DMFS (hazard ratio (HaR): 1.01 (1.00–1.02), *p* = 0.116). But for OS, an increment of 1% of tumour cells expressing Ki-67 increased by 1% the odds of death (HaR: 1.01 (1.00–1.02), *p* = 0.009). The optimal cut-offs retrieved by the analysis were >11 and >12 for DMFS and OS, respectively.

Posteriorly, it was tested whether there is a prognosis value of the semi-quantitative expression of HRs and what the optimal cut-off points are. However, 41 patients were classified in category 2+ of the ordinal (cross) system, which has a very broad expression (10%–60% of expressing cells) and generates inconsistencies for semi-quantitative reclassification. Furthermore, nine patients had no quantitative expression either in percentage or in the ordinal system. For this reason, these 50 patients were not included in further analyses. Thus, only 445 patients with luminal tumours were included in subsequent analyses.

For DMFS, the PR semi-quantitative expression (HaR: 0.83 (0.70–0.99), *p* = 0.035) was significant, meaning a 17% risk decrease by an increment of 1+. The composite semi-quantitative expression of hormone receptors (cHR) showed only a trend for OS (HaR: 0.90 (0.80–1.01), *p* = 0.064), meaning a 10% risk decrease by an increment of 1 point. The optimal cut-offs retrieved by the analysis were >1 and >4 for PR and cHR, respectively.

For OS, the PR semi-quantitative expression (HaR: 0.81 (0.68–0.96), *p* = 0.018) and cHR (HaR: 0.89 (0.79–1.00), *p* = 0.046) were significant, meaning a 19% and 11% risk decrease by an increment of 1 point by each system, respectively. The optimal cut-offs retrieved by the analysis were >1 and >4 for PR and cHR, respectively.

Although with a moderately strong correlation (Spearman’s rho: 0.398, *p* < 0.005) and association (Pearson’s *χ*^2^: 176.768, *p* < 0.005) between ER and PR, the semi-quantitative expression of ER was not significant for any outcome.

As the results were obtained through the analysis of variables that can violate the assumption of risk proportionality, they were categorised according to the cut-off points obtained in the previous analysis, and proportionality was tested using a KM curve and Log-Rank test. Cut-off points >12, >1 and >4 were selected for Ki-67, PR and cHR, respectively, as a function of significance in the results of previous analyses.

The cHR cut-off enabled the inclusion of three additional patients due to the single expression of ER on their tumours, totalising 448 luminal patients included in further analysis. The clinical data of the 572 patients, 448 with luminal tumours and 124 with triple-negative tumours, for further analysis from this point on are presented in Table 2.

**Figure 1.**
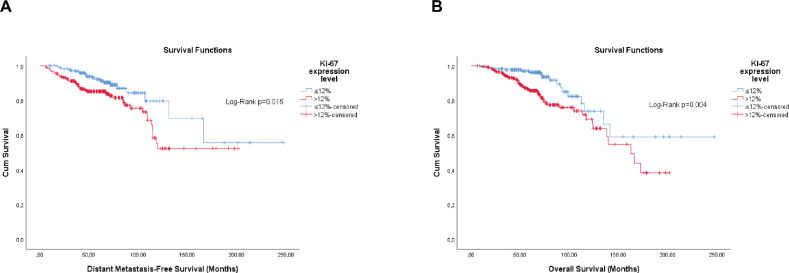
Cumulative survival curves by the KM estimator according to the Ki-67 expression level in luminal breast cancer patients (*n* = 448). (a): DMFS. (b): OS.

**Figure 2.**
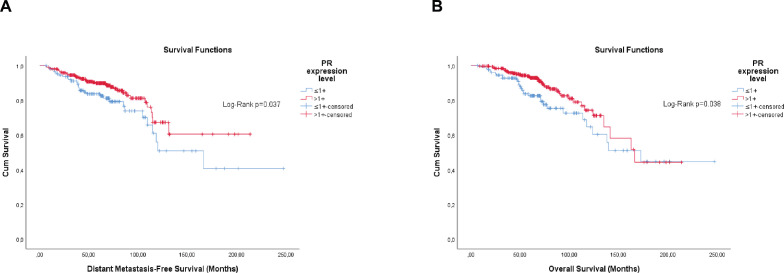
Cumulative survival curves by the KM estimator according to the semi-quantitative expression level of the PR in luminal breast cancer patients (*n* = 448). (a): DMFS. (b): OS. Legend: PR – Progesterone receptor.

**Figure 3.**
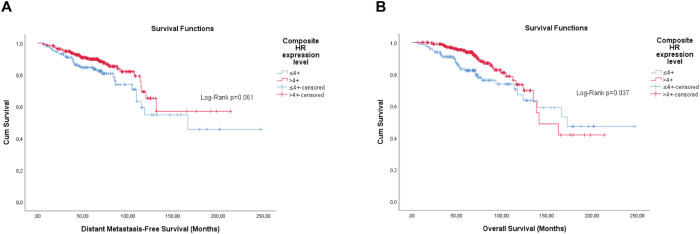
Cumulative survival curves by the KM estimator according to the cHR in luminal breast cancer patients (*n* = 448). (a): DMFS. (b): OS. Legend: cHR – Composite semi-quantitative expression of hormone receptors.

Overall, the categorisation of Ki-67, the semi-quantitative expression of PR and the composite of both hormone receptors showed significance, or were close, for both outcomes (Figures 1–3).

**Figure 4.**
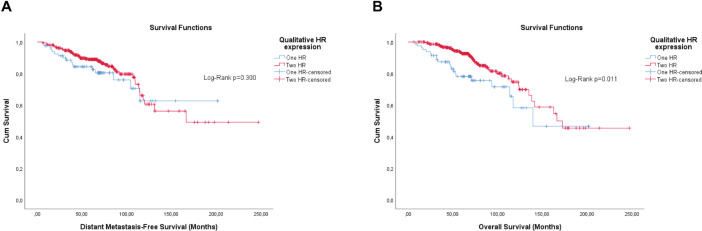
Cumulative survival curves by the KM estimator according to the qualitative expression of hormone receptors in luminal breast cancer patients (*n* = 448). (a): DMFS. (b): OS.

**Figure 5.**
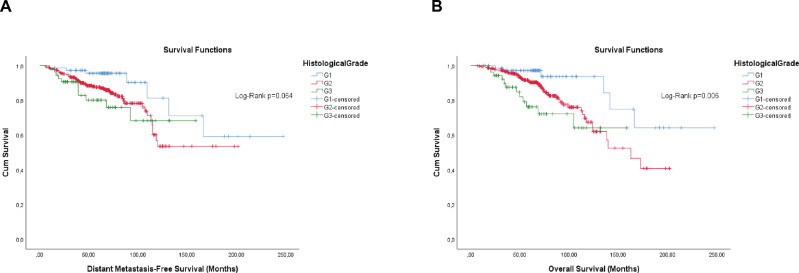
Cumulative survival curves by the KM estimator according to the histological grade in luminal breast cancer patients (*n* = 448). (a): DMFS. (b): OS. Legend: G1 – Well differentiated; G2 – Moderately differentiated; G3 – Poorly differentiated.

The qualitative expression of hormone receptors and the histological grade also have prognosis values. Therefore, they were evaluated by the KM method. Unlike the semi-quantitative expression, the qualitative expression of hormone receptors was only significant for OS (Figure 4). The histological grade also showed significance, or was close, for both outcomes (Figure 5).

Differently from pathological factors (*T* and *N* – data not displayed), the risks remain proportional during the first 60 months (5 years), with changes at later times and intersections being observed close to 120 months (10 years) for some factors (Figures 1–5). Therefore, time-dependent analysis was carried out using an interaction term between the categorised times of observation and the variables of interest.

For DMFS, only the semi-quantitative expressions of PR or cHR resulted in being independent after correction by other factors by stepwise and insert methods, respectively (Table 3).

For OS, the stepwise model included both Ki-67 and qualitative expression of HR as significant (Table 4). Due to strong to very strong correlations between semi-quantitative PR expression and cHR (Spearman’s rho: 0.802, *p* < 0.005); semi-quantitative PR expression and qualitative HR expression (Spearman’s rho:0.553, *p* < 0.005); and between cHR and qualitative HR expression (Spearman’s rho: 0.667, *p* < 0.005), the second model included the factor with the lowest *p*-value (cHR), which resulted in being significant after covariation (Table 4); the analysis including semi-quantitative PR expression showed significance after covariation (adjusted HaR: 0.465 (0.276–0.781), *p* = 0.004) and Ki-67 expression (adjusted HaR: 1.803 (1.012–3.210), *p* = 0.045).

### Stratified analysis allowing the identification of risk modification factors

Previous analyses resulted in the inclusion of different factors according to outcomes. However, histological grade and Ki-67 proliferative index are known risk factors in patients with luminal breast cancer. Aiming at a greater segregation of risks, stratified (hierarchical) analyses were carried out to study the effect of modification by other variables. As the semi-quantitative expressions of PR and cHR were independent variables for both outcomes, the hierarchy started with them.

Starting with the semi-quantitative PR expression level, both Ki-67 and histological grade showed some stratification only in patients with tumours classified as ≤1+. In preliminary analyses, G2 and G3 showed higher overlap for both outcomes. Therefore, these categories were grouped. The Ki-67 level showed significance for both DMFS (Log-Rank; *p* = 0.003) and OS (Log-Rank; *p* = 0.007), and histological grade for both DMFS (Log-Rank; *p* = 0.022) and OS (Log-Rank; *p* = 0.012) as well. As no interaction was observed (*p* > 0.05), conventional Cox regression was carried out with the stepwise method. The histological grade (G2/G3 versus G1) resulted in being independent for OS (adjusted HaR: 7.604 (1.007–57.405), *p* = 0.049), but Ki-67 (>12% versus ≤12%) for DMFS (adjusted HaR: 4.263 (1.331–13.652), *p* = 0.015).

Starting with cHR, both Ki-67 and histological grade showed some stratification only in patients with tumours classified as ≤4+. In preliminary analyses, G2 and G3 showed higher overlap for both outcomes. Therefore, these categories have been grouped. The Ki-67 level showed significance for both DMFS (Log-Rank; *p* = 0.020) and OS (Log-Rank; *p* = 0.020), and histological grade for both DMFS (Log-Rank; *p* = 0.047) and OS (Log-Rank; *p* = 0.017) as well. As no interaction was observed (*p* > 0.05), conventional Cox regression was carried out with the stepwise method. The histological grade (G2/G3 versus G1) resulted in being independent for both DMFS (adjusted HaR: 4.506 (1.206–16.827), *p* = 0.025) and OS (adjusted HaR: 7.601 (1.655 – 34.914), *p* = 0.009).

According to the first results, cHR showed consistence as an independent factor for both OS and DMFS. Thus, it is possible to conclude that it would be the best independent risk factor to start hierarchisation. Thus, it was further analysed whether Ki-67 could result in further risk discrimination in these patients, but no risk stratification was obtained. After checking for proportionality assumption by KM curves, time-dependent Cox regression showed that the first two categories (cHR > 4+ and cHR ≤4+ G1) have similar prognosis values for both DMFS (adjusted p=0.454) and OS (adjusted *p* = 0.290), thus they were grouped in a new two-level variable (cHR > 4+/ cHR ≤4+ G1 and cHR ≤4+ G2/G3).

Because histological grade is a known risk factor for luminal breast cancer patients, another hierarchical risk stratification approach was carried out starting with this variable. After checking for proportionality assumption by KM curves, time-dependent Cox regression showed that both semi-quantitative PR expression (adjusted HaR: 0.341 (0.182–0.639), *p* = 0.001) and cHR (adjusted HaR: 0.328 (0.180–0.597), *p* < 0.0005) are independent factors for DMFS, but mutually exclusive, and only for G2. Similarly, time-dependent Cox regression showed, again, that both semi-quantitative PR expression (adjusted HaR: 0.327 (0.174–0.614), *p* = 0.001) and cHR (adjusted HaR: 0.385 (0.201–0.737), *p* = 0.004) are independent factors for DMFS, but mutually exclusive, and only for G2. No further stratification was obtained by Ki-67.

By time-dependent Cox multivariable regression, it was possible to observe that in both classifications the two first levels have similar DMFS and OS, and the last two levels have similar DMFS and OS, but different from the first two (data not displayed). Thus, grouping was possible in two-level categories for semi-quantitative PR expression (G1/G2 PR>1+ and G2 PR ≤1+/G3) and cHR (G1/G2 cHR>4+ and G2 cHR ≤4+/G3).

The last approach was carried out starting with Ki-67 levels. For Ki-67>12%, both semi-quantitative PR expression (adjusted HaR: 0.326 (0.179–0.594), *p* < 0.0005) and cHR (adjusted HaR: 0.414 (0.229–0.823), *p* = 0.004) are significant for DMFS by time-dependent Cox regression. Again, for Ki-67>12%, both semi-quantitative PR expression (adjusted HaR: 0.387 (0.215–0.700), *p* = 0.002] and cHR (adjusted HaR: 0.439 (0.234–0.823), *p* = 0.010) are significant for OS by time-dependent Cox regression. No risk stratification was produced in Ki-67≤12% tumours or by histological grade in further analysis. By time-dependent Cox regression models, it was possible to observe similar prognosis factors by the first two levels of both classifications for DMFS and OS, enabling further clustering.

Regarding the qualitative expression of hormone receptors, stratified (hierarchical) analyses, whether based on this factor or others, did not show potential to segregate risks, since analysis by KM and Log-Rank test showed very high *p*-values.

### Identification of patients at risk similar to those with triple-negative tumours

Previous analyses have shown stratification in different ways and involving different factors. However, there is high collinearity between some of these factors. The semi-quantitative expression classifications of PR and both cHR similarly rank most patients (Cohen’s Kappa: 0.798, *p* < 0.005). There is correlation between histological grade and Ki-67 (Spearman’s rho: 0.317, *p* < 0.005), with a moderate association between histological grade and the Ki-67 cut-off (Pearson’s *χ*^2^: 27.244, *p* < 0.005).

None of the classifications by stratification shows 100% agreement with the other. For this reason, all were analysed. As the subsequent classification by cHR and histological grade followed the order of factors found in the initial analysis, its value in multivariable analysis has always been calculated in further analysis.

Triple-negative cancer patients were added as the third level to each category to identify the best risk stratification. As a comparison, the classification of tumours as luminal A and B was according to the expression of PR and Ki-67 (luminal A: PR≥20% and Ki-67<14%; luminal B: PR<20% and/or Ki-67±14%). As there was 100% agreement between the cut-off points obtained (>12%) and that found in the literature (>14%) (Cohen’s Kappa: 1.000, *p* < 0.005), the former was maintained. As most medical records did not report the percentage of PR-expressing tumour cells, this cut-off point was replaced by PR>1+. Alternatively, the same classification was carried out but with a modified Ki-67 cut-off to 20% as per the modern guidelines.

The stepwise model retained the hierarchical risk stratification by Ki-67 and PR (Tables 5 and 6). Although the classifications similar to those currently accepted are significant, the classifications previously obtained proved to be superior and capable of identifying patients with luminal breast cancer whose prognosis is similar to that of patients with triple-negative breast cancer (Tables 5 and 6).

However, the first approach resulted in a good risk stratification and identification of luminal breast tumours patients with prognosis similar to those with triple-negative breast tumours for both DMFS (cHR ≤4+ G2 or G3 versus triple-negative, adjusted HaR: 0.703 (0.415–1.189), *p* = 0.189) and OS (cHR ≤4+ G2 or G3 versus triple-negative, adjusted HaR: 0.662 (0.403–1.088), *p* = 0.104).

The second approach also resulted in a good risk stratification and identification of luminal breast tumours patients with prognosis similar to those with triple-negative breast tumours for both DMFS (G2 PR ≤1+or G3 versus triple-negative, adjusted HaR: 0.650 (0.385–1.095), *p* = 0.106), but with almost a difference for OS (G2 PR ≤1+ or G3 versus triple-negative, adjusted HaR: 0.628 (0.387–1.019), *p* = 0.059).

Similarly, the third approach resulted in a good risk stratification and identification of luminal breast tumour patients with prognosis similar to those with triple-negative breast tumours for DMFS (G2 cHR ≤4+ or G3 versus triple-negative, adjusted HaR: 0.654 (0.394–1.086), *p* = 0.101), but not for OS (G2 cHR ≤4+ or G3 versus triple-negative, adjusted HaR: 0.603 (0.373–0.973), *p* = 0.038).

Finally, the fifth approach resulted in a good risk stratification and identification of luminal breast tumour patients with prognosis similar to those with triple-negative breast tumours for both DMFS (Ki-67>12% cHR≤4+ versus triple-negative, adjusted HaR: 0.691 (0.406–1.174), *p* = 0.172) and OS (Ki-67>12% cHR≤4+ versus triple-negative, adjusted HaR: 0.695 (0.416–1.161), *p* = 0.165).

**Figure 6.**
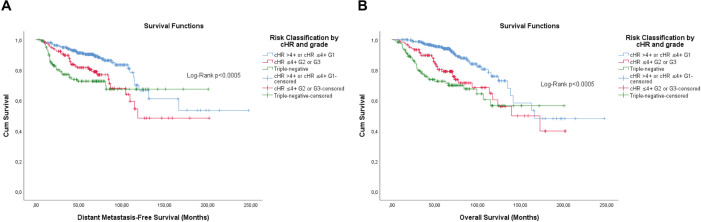
Cumulative survival curves by the KM estimator according to the subsequent risk stratification by cHR and histological grade. (a): DMFS. (b): OS. A total of 572 patients were included. Legend: cHR – Composite semi-quantitative expression of hormone receptors; G1 – Well differentiated; G2 – Moderately differentiated; G3 – Poorly differentiated.

**Figure 7.**
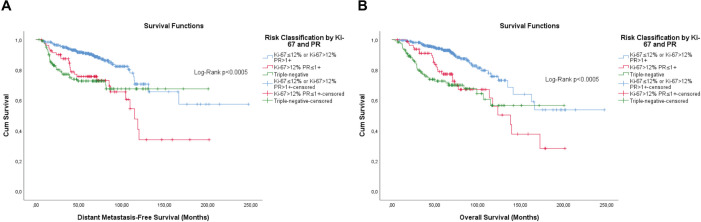
Cumulative survival curves by the KM estimator according to the subsequent risk stratification by Ki-67 and semi-quantitative expression of the progesterone receptor (PR). (a): DMFS. (b): OS. A total of 572 patients were included.

**Figure 8.**
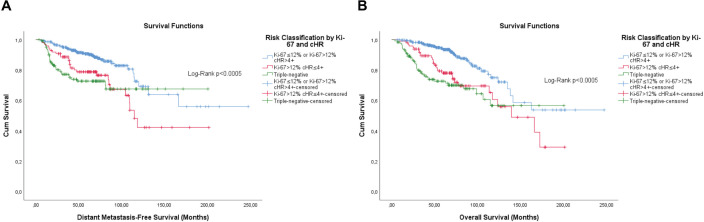
Cumulative survival curves by the KM estimator according to the subsequent risk stratification by Ki-67 and cHR. (a): DMFS. (b): OS. A total of 572 patients were included.

The KM curves of the three fittest approaches are shown in Figures 6–8.

### Factors associated with increased risk of metastasis and death in patients of adjuvant schema

Due to the impact that neoadjuvant treatment has on biomarker expression and prognosis, analyses were carried out on the subset of patients who received only adjuvant regimens to test for similar stratifications (*n* = 358). Unlike the wide variety of possibilities for hierarchies in the total set of luminal breast cancer patients included, stratification based on the histological degree made it possible to discriminate between subgroups with similar prognoses. At the second level of hierarchy, it was possible to observe that Ki-67 promoted prognosis discrimination in patients with grade G1/G2 disease for DMFS (Log-Rank; *p* = 0.022) and OS (Log-Rank; *p* = 0.044), being the only significant biomarker in multivariable analysis for DMFS (adjusted HaR: 1.998 (1.019–3.915), *p* = 0.043), but not significant for OS (adjusted HaR: 1.635 (0.838–3.193), *p* = 0.150).

Next, we tested whether the semi-quantitative expression of hormone receptors can lead to better discrimination, and a positive response was observed for both PR (DMFS Log-Rank, *p* = 0.004; OS Log-Rank, *p* = 0.036) and cHR (DMFS Log-Rank, *p* = 0.006; OS Log-Rank, *p* = 0.015), but only in cases with Ki-67 > 12%. It was observed that PR and cHR are independent and superior to DMFS (adjusted HaR: 0.337 (0.155–0.730), *p* = 0.006) and OS (adjusted HaR: 0.417 (0.189–0.921), *p* = 0.031), with only a trend for OS (adjusted HaR: 0.498 (0.234–1.058), *p* = 0.070) and DMFS (adjusted HaR: 0.507 (0.244–1.053), *p* = 0.069), respectively.

**Figure 9.**
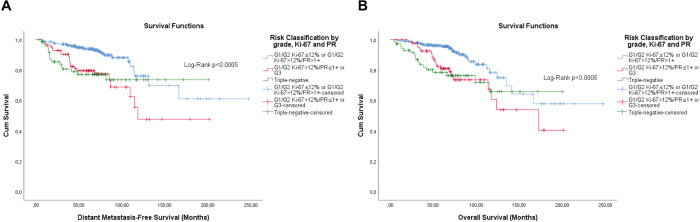
Cumulative survival curves by the KM estimator according to the subsequent risk stratification by grade, Ki-67 and semi-quantitative expression of the progesterone receptor (PR). (a) DMFS. (b): OS. A total of 431 patients of adjuvant schema were included.

**Figure 10.**
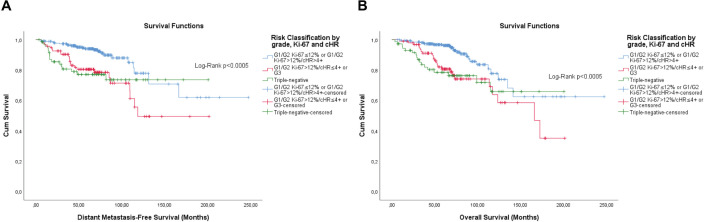
Cumulative survival curves by the KM estimator according to the subsequent risk stratification by grade, Ki-67 and semi-quantitative expression of both hormone receptors (cHR). (a): DMFS. (b): OS. A total of 431 patients of adjuvant schema were included.

Subsequently, multivariable analyses were carried out to test the possibility of performing clusters. Both risk classification strategies proved to be significant for DMFS and OS. Through repeated contrast, it was possible to observe a clear distinction between the second and third groups for both strategies in the two outcomes tested (data not shown). Therefore, the first two and last two groups were grouped. These classifications have a high, but incomplete, degree of agreement (Cohen’s Kappa: 0.876, *p* < 0.0005) with each other. Figures 9 and 10 show the survival curves including triple-negative patients also on an adjuvant regimen only.

### Adjustments for treatments do not change the prognosis value of risk ratings

Finally, it was analysed whether treatments could be a confounding factor. But first, it was tested in which context chemotherapy results results in a better prognostic for patients with luminal cancer in order to improve the classification of systemic treatment.

Patients with luminal breast cancer from an adjuvant regimen were segregated according to biomarkers and it was evaluated whether chemotherapy implies a better prognosis, since analyses with all patients did not demonstrate a potential benefit of chemotherapy. As it may be an effect modification, the * operation was carried out between pN (N0, N1 and N2/N3) and chemotherapy (No and Yes).

Among the biomarkers, the only one that showed ‘predictive’ value was Ki-67. It was possible to observe that pN is the main factor associated with lower DMFS. It was observed that pN2/N3 is a high-risk factor associated with DMFS (versus pN0, adjusted HaR: 20.778 (6.532–66.092) *p* < 0.0005) and chemotherapy modifies the effect on pN2/N3 (adjusted HaR: 0.093 (0.010–0.833) *p* = 0.034), but not on pN1

(*p* = 0.610). Such an effect modification was also observed regarding OS for pN2/N3 (adjusted HaR: 0.103 (0.014–0.743) *p* = 0.024), but not for pN1 (*p* = 0.858).

As chemotherapy was not significant in the models to validate its interaction with lymph node metastasis, subgroup analyses were carried out. In fact, chemotherapy was observed to be the only factor associated with OS (HaR: 0.263 (0.092–0.752) *p* = 0.013) in patients with pN2/N3 and Ki-67>12% disease, with a trend for DMFS in association to radiation therapy for DMFS (adjusted HaR: 0.346 (0.115 – 1.045) *p* = 0.060).

Regarding risk classifications, only the last two showed a ‘predictive’ value of benefit from chemotherapy, but only for OS. Patients with pN2/N3 disease and tumors classified as G1/G2 Ki-67>12%/cHR≤4+ or G3 (adjusted HaR: 0.043 (0.002–0.758) *p* = 0.032), or G1/G2 Ki-67>12%/PR≤1+ or G3 disease (adjusted HaR: 0.039 (0.002–0.725) *p* = 0.030) showed effect modification by chemotherapy, but again, without chemotherapy as significant in the models. However, a trend of benefit of chemotherapy was observed in the first group only by subgroup analysis according to pN2/N3 disease (HaR: 0.252 (0.060–1.064) *p* = 0.061).

Therefore, systemic treatment was re-categorised based on the previous results, assigning appropriate treatment classifications to patients whenever they received hormone therapy, and they received chemotherapy if and only if they had pN2/3 disease and Ki-67>12%, which resulted superior to the initial classification, according to the St Gallen guidelines.

Finally, models were adjusted to include adjuvant regimen-only treatments. For this, time from the end of the adjuvant therapies (chemotherapy or radiotherapy) until the event or censorship was considered; patients who developed events during adjuvant therapy (chemotherapy or radiotherapy) were excluded. A total of 419 patients were included in the analyses. Two models using the two-block approach were built. The first model included all risk ratings in the first block; the second model was designed excluding the first block and the risk classification obtained in the first model.

The stratifications with Ki-67 in the first hierarchical level continued to be statistically superior to the others, even compared to those described in the literature, with change from PR, at the second hierarchical level referring to DMFS, to cHR, at the second hierarchical level, and to OS (Tables 7 and 8). Nonetheless, the second model for both DMFS and OS agreed that the sequential risk stratification by histological grade, Ki-67 and cHR as independent factors is superior to the others (Tables 7 and 8).

Risk stratifications involving only histological grade and semi-quantitative expression of hormone receptor(s), regardless of the order, did not result in good identification of patients with luminal cancer whose prognoses are similar to triple-negative. Regarding DMFS, the second category of subsequent stratification by cHR and grade, grade and PR, and grade and cHR showed a better prognosis than triple-negative tumours with *p*-values of 0.034 (adjusted HaR: 0.415 (0.184–0.937)), 0.064 (adjusted HaR: 0.473 (0.214–1.045)) and 0.050 (adjusted HaR: 0.456 (0.209–0.999)), respectively. Regarding OS, the second category of subsequent stratification by cHR and grade, grade and PR, and grade and cHR showed similar prognosis than triple-negative tumours with *p*-values of 0.336 (adjusted HaR: 0.704 (0.344–1.440)), 0.374 (adjusted HaR: 0.721 (0.350–1.484)) and 0.333 (adjusted HaR: 0.706 (0.349–1.429)), respectively.

## Discussion

The identification of luminal breast cancer cases with a high risk of metastasis development is critical for a therapeutic decision [10, 17]. However, the only prognostic and predictive test currently available with grade 1A evidence for both therapeutic decision and staging is the Oncotype Dx genomic test [49–51], which is expensive.

Studies have shown consecutive risk stratification in luminal cancer cases by Ki-67 [9, 10], with subsequent prognostic gain by segregation according to the semi-quantitative expression of PR [52], but the unification with the histological grade remains a difficulty [10, 17, 38]. Ehinger *et al* [53] observed that, in patients with luminal ER^+^ tumours, the semi-quantitative expression of PR and Ki-67 discriminates prognosis only in moderately differentiated (G2) tumours. Furthermore, although other studies show the Ki-67 index as a risk segregation criterion in G2 tumours [12, 54], Liang *et al* [55] observed similar prognosis between patients with G3 tumours and high Ki-67 expression in G1 and G2 tumours regarding recurrences. We were able to incorporate these three factors but only in patients of adjuvant regimen, with consecutive hierarchical levels by histological grade, Ki-67 and, finally, the semi-quantitative expression of both hormone receptors, or just of the progesterone receptor. Even so, the stratification path that the initial analysis pointed to was another, promoting consecutive stratification by cHR and later by histological grade, showing similar prognosis between G2 and G3 diseases with low expression of both hormone receptors. Notwithstanding, this stratification did not result in a good risk segregation for DMFS in patients of adjuvant schema as we observed for consecutive stratification by histological grade, Ki-67 proliferation index and, finally, cHR.

Interestingly, all classifications obtained in the analysis turned out to be superior to the currently accepted luminal breast cancer classification (luminal A and B) [10, 17]. However, due to the limitation in reporting the expression of hormone receptors, only one approximation was made. In fact, the used PR >1+ category represents tumours with more than 10% expressing cells [46, 47], which is biased while making proper comparisons.

Even though the classification incorporating PR levels proved to be interesting, the classifications involving cHR respected some theoretical foundations. For example, luminal tumours expressing only the PR are more aggressive, being a factor of poorer prognosis for relapses and death, even when compared to ER^+^/PR^-^ cases [56–60]. Additionally, a recent meta-analysis demonstrated that low ER-expressing (1%–10%) tumours have an impaired endocrine response [61], a typical characteristic of more aggressive luminal tumours [62]. Thus, classification considering cHR naturally classified tumours with low ER expression and expression of only one hormone receptor as similar, although this approach is not largely validated.

Another important theoretical factor is how the interaction between the receptors takes place. In oestrogen-responsive tumours, PR is expressed as a response to the latter via the ER [63], with subsequent modification of the ER interaction network and expression of different target genes in a feedforward fashion [64]. In line with this, we observed that low ER-expressing tumours are likely to express low PR levels, whereas high ER-expressing tumours express high levels of PR, suggesting that this effect probably occurs in a dose-dependent manner. Thus, the semi-quantitative expression of both HRs could respond for part of the highly intricate biology of breast cancers, as we observed for both studied outcomes, and it respects the fact that the qualitative expression of HR is a more important prognosis factor for OS.

Genetic studies with different panels show segregation of the histological grade into two genetic grades (low and high), which have a higher prognosis value than the histological grade [65]. Interestingly, there is a high degree of agreement between the histological grade G1 and the low genetic grade and the histologic grade G3 with the high genetic grade, but high segregation in the histological grade G2 between these two genetic grades [66, 67]. In fact, the histological grade G2 is the classification with the greatest disagreement among the three [68–70], and some studies suggest that the proliferative index can promote a prognosis distinction precisely in G2 [12, 54].

A study by Sotiriou *et al* [71] observed that low and high genetic grades can segregate patients with PR^+^ tumours according to endocrine responsiveness and cell proliferation, whereas the low genetic grade has lower proliferation and greater endocrine responsiveness. We observed that the semi-quantitative expression made efficient segregation of the risk factor, but only in G1/G2 patients with a high Ki-67 proliferative index, which finally defined the luminal patients in two groups, which is in harmony with the study by Arima *et al* [72], in which they observed that in luminal patients (HER2^-^) the expression of PR does not add a prognosis factor in patients with low proliferative index by Ki-67, but the high expression of RP (≥20%) partially counterbalances the negative effect of the high proliferative index by Ki-67, as we observed in this study.

The low sample number of patients with G1 and G3 tumours prevented us from evaluating whether these other two factors would cause any segregation of risks. However, Ehinger *et al* [53] observed that patients with luminal breast cancer (ER^+^/HER2^-^) of histological grade G3 have a similar prognosis factor, regardless of whether they are classified as luminal A or B based on the Ki-67 proliferative index and qualitative PR expression. In fact, there is a consensus that high histological grade (G3) is a factor of worse prognosis in luminal A patients [10].

Furthermore, an expression of both hormone receptors, not just PR, correlates with the Oncotype Recurrence Score [35, 37, 73], and has a prognosis value for distant metastases by the IHC4 predictive model [74], which has a similar prognosis value to Oncotype Dx for early metastases (up to 5 years) [74–76]. Thus, there is more than enough scientific support to analyse the semi-quantitative expression of both receptors as a prognosis factor, and it is versatile in allowing both risk segregation for distant metastasis and potential staging for overall survival, unlike the qualitative expression which is a known staging factor for overall survival [25], as we also observed in the initial analyses.

In this study, the histological grade and the Ki-67 proliferative index proved to be mutually exclusive in several analyses. One possibility is the correlation that exists between them [77], because the mitotic index is one of the classification criteria of the Nottingham system [11, 45]. Another possibility is the changes that neoadjuvant chemotherapy causes in the tumour microenvironment and in the selection of subclones with different phenotypes, causing changes in the expression of hormone receptors, mitotic index and Ki-67 [78–83], which may explain why it was possible to incorporate histological grade and Ki-67 only in patients on the adjuvant regimen, but not in the group including those on the neoadjuvant regimen.

Although showing the superiority of prognosis segregation in the incorporation of the three analysed parameters (histological grade, Ki-67 and semi-quantitative expression of hormone receptors), having presented other stratification models is interesting for some situations. For example, the treatment guidelines approved for the public health system in Brazil make the assessment of histological grade and identification of the three important tumour receptors mandatory, but not Ki-67 [84].

An interesting factor related to the study of these biomarkers is the potential benefit of chemotherapy. Although the focus was not the discovery of a predictive classification system for the benefit of chemotherapy, it was possible to observe that Ki-67 is the best predictive factor for this response, but only in patients with pN2/N3 disease. This is in agreement with data in the literature, which show that Ki-67 is the best predictor of response to chemotherapy in patients with luminal breast cancer [85, 86]. Interestingly, we could observe that patients with G1/G2 Ki-67>12%/cHR≤4+ or G3 disease show a modification of effect by chemotherapy when there is the involvement of four or more lymph nodes (pN2/N3) for OS. In fact, there is evidence that patients with tumours of good biological risk (low grade, low proliferation and increased expression of hormone receptors) have little or no benefit from chemotherapy, even in the presence of metastatic lymph nodes, but the contrary is true [62]. Thus, this hierarchical classification can even help in clinical decision-making for treatment.

This study has several limitations, as the long elapsed period, which implies the incorporation of new therapies. Other limitations are the possibility of IHC analysis artefacts [4], which we did not have access to, and the reproducibility and feasibility of different antibody clones [4, 87–90], which were used by the Institution, that could dampen our results. Also, the low number of patients representing certain histopathological and immunohistochemical features, such as G1 and G3 tumours, and tumours with low expressions of hormone receptors and/or only one hormone receptor led to results with very wide confidence intervals due to the low number of events observed in these categories.

Regarding Ki-67, we analysed only one dichotomisation, according to the statistical methodology used. However, it is known that there are multilevel categorisations of Ki-67 that better relate to the development of relapses and response to chemotherapy [17, 38, 62, 85, 86].

In addition, we could not observe the median time in most analyses, being observed practically only for the second category of classification of luminal patients of all reclassifications, but not for the first category or patients with triple-negative tumours, which makes the results on prognosis factors dependent on the survival curves of this cohort.

But the main limitation is the classification of the semi-quantitative expression of hormone receptors. Due to the lack of standardisation of the semi-quantitative expression of these, which has recently been incorporated into the guidelines of the public health system in Brazil [84], we needed to standardise the reports to the ordinal system of crosses by Sannino and Shousha [46, 47]. This leads to the loss of information that more refined systems provide, such as Allred or H-score systems [91, 92]. Despite these limitations, our results do not greatly contrast with those in the literature.

A study with a large cohort and improved immunohistochemical data (percentage and intensity) could show the validity of the presented approach and whether it is possible to integrate histological grade and Ki-67, along with semi-quantitative expression of hormone receptors, to identify patients at high risk of developing distant metastases and death.

## Conclusion

The different risk stratifications prove to be advantageous when there are limitations of information obtained by IHC techniques according to clinical practices adopted in regions with few resources, such as in low- and middle-income countries. The fact that risk stratification with better identification of high-risk patients, similar to those with triple-negative cancer, was generated by the incorporation of all the variables included attests to the need to include a detailed analysis of these factors in the routines and guidelines of countries such as Brazil.

## List of abbreviations

AJCC: American Joint Committee on Cancer
cHR: Composite semi-quantitative expression of hormone receptors
DMFS: Distant metastasis-free survival
ER: Oestrogen receptor
G1: Well-differentiated histological grade
G2: Moderately differentiated histological grade
G3: Poorly differentiated histological grade
HR: Hormone receptor
HaR: Hazard ratio
IHC: Immunohistochemistry
KM: Kaplan–Meier estimator curve
*N*
: Lymph node metastasis, categorisation by AJCC guidelines
OS: Overall survival
PR: Progesterone receptor
*T*
: Tumour size, categorisation by AJCC guidelines

## Conflicts of interest

The authors declared no conflicts of interest.

## Funding statement

The authors received no financial support for the research, authorship and/or publication of this article.

## Author contributions

FACL conceived the study. FACL, ECM, CPN, LAM and PFRD designed the methodology and collected data. FACL carried out the data analysis. RMF, RAR and MJBS supervised and validated the data collection and analysis. FACL wrote the initial manuscript. All authors reviewed and approved the final draft.

## Data availability

The data generated for this study is publicly available at DOI: 10.17632/k4ycfhbmbx.3

## Figures and Tables

**Table 1.** Clinical data of included patients in initial analysis (*n* = 619).

Variable	*N* (%)	Median (min.– max.)
Time to distant metastasis	619 (100)	62.50 months (3.67–247.40)
Time of observation	619 (100)	64.90 months (3.67–247.40)
Age	619 (100)	56 years (26–92)
Age		
<70 years	520 (84.0)	
≥70 years	99 (16.0)	
Age		
≤40 years	61 (9.9)	
>40 years	558 (90.1)	
Events of distant metastasis	112 (18.1)	
Events of all-cause death	112 (18.1)	
Surgery approach		
Breast-conserving	338 (54.6)	
Mastectomy	281 (45.4)	
Surgical margin		
Negative	571 (92.2)	
Positive	48 (7.8)	
*T*		
*T*1	194 (31.3)	
*T*2	265 (42.8)	
*T*3	103 (16.6)	
*T*4	57 (9.2)	
*N*		
*N*0	331 (53.5)	
*N*1	183 (29.6)	
*N*2	75 (12.1)	
*N*3	30 (4.8)	
Histological grade		
G1	87 (14.1)	
G2	401 (64.8)	
G3	131 (21.2)	
Qualitative hormone receptor expression		
No (triple-negative)	124 (20.0)	
One (ER^-^/PR^+^)	20 (4.2)	
One (ER^+^/PR^-^)	62 (9.1)	
Two (ER^+^/PR^+^)	413 (66.7)	
Ki-67 expression (luminals only)	495	20% (1–97)
Hormonal therapy (luminals only)		
No	16/495 (3.2)	
Yes	479/495 (96.8)	
Chemotherapy		
No	140 (22.6)	
Neoadjuvant	150 (24.2)	
Adjuvant	329 (53.2)	
Systemic treatment		
Inadequate	59 (9.5)	
Adequate	560 (90.5)	
Local radiotherapy		
Inadequate	45 (7.3)	
Adequate	574 (92.7)	

Legends: G1 – Well differentiated; G2 – Moderately differentiated; G3 – Poorly differentiated; *N* – Lymph node metastasis; *T* – Tumour size

**Table 2.** Clinical data of included patients in most analysis (*n* = 572).

Variable	*N* (%)	Median (min.– max.)
Time to distant metastasis	572 (100)	62.28 months (3.67–247.40)
Time of observation	572 (100)	65.35 months (3.67–247.40)
Age	572 (100)	56 years (26–92)
Age		
<70 years	481 (84.1)	
≥70 years	91 (15.9)	
Age		
≤40 years	58 (10.1)	
>40 years	514 (89.9)	
Events of distant metastasis	105 (18.4)	
Events of all-cause death	105 (18.4)	
Surgery approach		
Breast-conserving	316 (55.2)	
Mastectomy	256 (44.8)	
Surgical margin		
Negative	528 (92.3)	
Positive	44 (7.7)	
*T*		
*T*1	178 (31.1)	
*T*2	243 (42.5)	
*T*3	96 (16.8)	
*T*4	55 (9.6)	
*N*		
*N*0	307 (53.7)	
*N*1	171 (29.9)	
*N*2	66 (11.5)	
*N*3	28 (4.9)	
Histological grade		
G1	77 (13.5)	
G2	372 (65.0)	
G3	123 (21.5)	
Qualitative hormone receptor expression		
No (triple-negative)	124 (21.7)	
One (ER^-^/PR^+^)	20 (4.6)	
One (ER^+^/PR^-^)	62 (9.7)	
Two (ER^+^/PR^+^)	366 (64.0)	
Ki-67 expression (luminals only)	448	20% (1 – 97)
Hormonal therapy (luminals only)		
No	15/448 (3.3)	
Yes	433/448 (96.7)	
Chemotherapy		
No	126 (22.0)	
Neoadjuvant	141 (24.7)	
Adjuvant	305 (53.3)	
Systemic treatment		
Inadequate	56 (9.8)	
Adequate	516 (90.2)	
Local radiotherapy		
Inadequate	40 (7.0)	
Adequate	532 (93.0)	

Legends: G1 – Well differentiated; G2 – Moderately differentiated; G3 – Poorly differentiated; *N* – Lymph node metastasis; *T* – Tumour size

**Table 3.** Univariable and multivariable time-dependent Cox analyses of factors associated with distant metastasis in luminal breast cancer patients (*n* = 448).

	Univariable		Multivariable			
Factor			Model 1^a^		Model 2^b^	
	HaR (95%CI)	*p*	HaR (95%CI)	*p*	HaR (95%CI)	*p*
Ki-67 expression						
≤12%	1		1		1	
>12%	1.750 (1.039–2.946)	0.035	1.192 (0.694–2.048)	0.525	1.140 (0.659–1.972)	0.640
Semi-quantitative PR expression						
≤1+	1		1			
>1+	0.623 (0.386–1.004)	0.052	0.440 (0.262–0.740)	0.002		
Semi-quantitative HR expression						
≤4+	1				1	
>4+	0.591 (0.371–0.942)	0.027			0.524 (0.316–0.867)	0.012
Qualitative HR expression						
One HR	1					
Two HR	0.720 (0.417–1.243)	0.238				
Histological grade		0.270				
G1	1					
G2	1.710 (0.767–3.815)	0.190				
G3	2.208 (0.841–5.800)	0.108				
Age						
≤40 years	1		1		1	
>40 years	0.459 (0.233–0.904)	0.024	0.400 (0.196–0.814)	0.011	0.438 (0.216–0.887)	0.022
*T*		<0.0005	0.002		0.003	
*T*1	1		1		1	
*T*2–*T*3	2.521 (1.259–5.050)	0.009	1.459 (0.706–3.014)	0.308	1.432 (0.689–2.976)	0.336
*T*4	9.283 (4.244–20.034)	<0.0005	3.889 (1.660–9.112)	0.002	3.566 (1.522–8.355)	0.003
*N*		<0.0005	<0.0005		<0.005	
*N*0	1		1		1	
*N*1	2.785 (1.364–5.688)	0.005	2.135 (1.020–4.471)	0.044	2.281 (1.091–4.771)	0.029
*N*2	10.242 (5.131–20.445)	<0.0005	7.347 (3.591–15.031)	<0.0005	6.900 (3.333–14.283)	<0.0005
*N*3	16.478 (7.418–36.599)	<0.0005	14.111 (5.993–33.229)	<0.0005	14.054 (5.928–33.319)	<0.0005
T_COV	0.946 (0.906–0.988)	0.013	0.624 (0.055–7.080)	0.525	0.609 (0.040–9.230)	0.720

a Two-block model: first with the Stepwise Forward Wald method with entry and exit *p*-values of <0.10 and <0.05, respectively

b Model including only significant (*p* < 0.05) factors of the univariable analysis or with known theoretical values

Legends: G1 – Well differentiated; G2 – Moderately differentiated; G3 – Poorly differentiated; HR – Hormone receptors; *N* – Lymph node metastasis; PR – Progesterone receptor; *T* – Tumour size

**Table 4.** Univariable and multivariable time-dependent Cox analyses of factors associated with all-cause death in luminal breast cancer patients (*n* = 448).

	Univariable		Multivariable			
Factor			Model 1^a^		Model 2^b^	
	HaR (95%CI)	p	HaR (95%CI)	p	HaR (95%CI)	*p*
Ki-67 expression						
≤12%	1		1		1	
>12%	2.467 (1.425–4.273)	0.001	2.051 (1.173–3.587)	0.012	1.753 (0.982–3.131)	0.058
Semi-quantitative PR expression						
≤1+	1					
>1+	0.530 (0.327–0.859)	0.010				
Semi-quantitative HR expression						
≤4+	1				1	
>4+	0.519 (0.321–0.840)	0.008			0.486 (0.286–0.827)	0.008
Qualitative HR expression						
One HR	1		1			
Two HR	0.534 (0.319–0.896)	0.017	0.487 (0.283–0.840)	0.010		
Histological grade		0.045			0.249	
G1	1				1	
G2	2.234 (0.943–5.292)	0.068			1.902 (0.769–4.703)	0.164
G3	3.601 (1.312–9.884)	0.013			2.493 (0.848–7.327)	0.097
Age						
<70 years	1		1		1	
≥70 years	1.874 (1.076–3.265)	0.027	1.908 (1.052–3.461)	0.011	2.051 (1.133–3.711)	0.018
*T*		<0.0005	0.041		0.023	
*T*1	1		1		1	
*T*2–*T*3	2.451 (1.225–4.903)	0.011	1.660 (0.797–3.459)	0.176	1.733 (0.836–3.592)	0.139
T4	6.642 (2.957–14.917)	<0.0005	3.046 (1.260–7.360)	0.013	3.382 (1.393–8.210)	0.007
*N*		<0.0005	<0.0005		0.001	
*N*0	1		1		1	
*N*1	2.285 (1.196–4.365)	0.012	1.668 (0.852–3.263)	0.135	1.552 (0.793–3.039)	0.199
*N*2	5.209 (2.694–10.070)	<0.0005	3.979 (2.002–7.911)	<0.0005	3.365 (1.690–6.699)	0.001
*N*3	6.691 (2.995–14.949)	<0.0005	3.871 (1.577–9.497)	0.003	3.656 (1.466–9.115)	0.005
T_COV	0.946 (0.905–0.990)	0.016	0.783 (0.319–1.922)	0.594	0.609 (0.040–9.230)	0.720

a Two-block model: first with the Stepwise Forward Wald method with entry and exit *p*-values of <0.10 and <0.05, respectively

b Model including only significant (*p* < 0.05) factors of the univariable analysis or with known theoretical values

Legends: G1 – Well differentiated; G2 – Moderately differentiated; G3 – Poorly differentiated; HR – Hormone receptors; *N* – Lymph node metastasis; PR – Progesterone receptor; *T* – Tumour size

**Table 5.** Univariable and multivariable time-dependent Cox analyses of factors associated with distant metastasis in breast cancer patients (*n* = 572).

	Univariable		Multivariable			
Factor			Model 1^a^		Model 2^b^	
	HaR (95%CI)	*p*	HaR (95%CI)	*p*	HaR (95%CI)	*p*
Hierarchical risk stratification by cHR and histological grade		<0.0005				
Triple-negative	1					
cHR >4+ or cHR ≤4+ G1	0.393 (0.248–0.624)	<0.0005				
cHR ≤4+ G2 or G3	0.772 (0.468–1.273)	0.310				
Hierarchical risk stratification by histological grade and PR expression		<0.0005				
Triple-negative	1					
G1 or G2 PR>1+	0.380 (0.237–0.610)	<0.0005				
G2 PR ≤1+or G3	0.743 (0.457–1.210)	0.233				
Hierarchical risk stratification by histological grade and cHR		<0.0005				
Triple-negative	1					
G1 or G2 cHR>4+	0.369 (0.229–0.595)	<0.0005				
G2 cHR ≤4+ orG3	0.745 (0.462–1.202)	0.288				
Hierarchical risk stratification by Ki-67 and PR		<0.0005				<0.0005
Triple-negative	1				1	
Ki-67≤12% or Ki-67>12% PR>1+	0.399 (0.254–0.627)	<0.0005			0.329 (0.206–0.526)	<0.0005
Ki-67>12% PR≤1+	0.909 (0.535–1.545)	0.726			0.810 (0.466–1.407)	0.454
Hierarchical risk stratification by Ki-67 and cHR		<0.0005				
Triple-negative	1					
Ki-67≤12% or Ki-67>12% cHR>4+	0.388 (0.246–0.613)	<0.0005				
Ki-67>12% cHR≤4+	0.870 (0.522–1.449)	0.592				
St Gallen classification (Former)		0.002				
Triple-negative	1					
Ki-67≤12% and PR >1+	0.359 (0.196–0.655)	0.001				
Ki-67>12% and/or PR ≤1+	0.553 (0.358–0.856)	0.008				
St Gallen classification (modern)		0.001		<0.0005		
Triple-negative	1		1			
Ki-67<20% and PR >1+	0.347 (0.196–0.613)	<0.0005	0.334 (0.186–0.598)	<0.0005		
Ki-67≥20% and/or PR ≤1+	0.583 (0.375–0.907)	0.017	0.452 (0.285–0.716)	0.001		
Age						
≤40 years	1		1		1	
>40 years	0.466 (0.275–0.788)	0.004	0.631 (0.366–1.087)	0.097	0.600 (0.348–1.036)	0.067
*T*		<0.0005		0.006		0.002
*T*1	1		1		1	
*T*2–*T*3	3.349 (1.766–6.351)	<0.0005	1.731 (0.882–3.398)	0.111	1.782 (0.917–3.466)	0.088
*T*4	10.855 (5.313–22.180)	<0.0005	3.299 (1.507–7.220)	0.003	3.602 (1.669–7.776)	0.001
*N*		<0.0005		<0.0005		<0.0005
*N*0	1		1		1	
*N*1	3.252 (1.885–5.613)	<0.0005	2.734 (1.548–4.829)	0.001	2.682 (1.525–4.717)	0.001
*N*2	8.038 (4.553–14.191)	<0.0005	6.513 (3.546–11.960)	<0.0005	6.427 (3.528–11.707)	<0.0005
N3	14.185 (7.377–27.279)	<0.0005	11.047 (5.439–22.436)	<0.0005	12.248 (6.048–24.806)	<0.0005
T_COV	0.943 (0.903–0.985)	0.008	0.577 (0.052–6.390)	0.654	0.591 (0.064–5.453)	0.643

a Two-block model: first with the Stepwise Forward Wald method with entry and exit *p*-values of <0.10 and <0.05, respectively, using published factors

b Two-block model: first with the Stepwise Forward Wald method with entry and exit *p*-values of <0.10 and <0.05, respectively

Legends: G1 – Well differentiated; G2 – Moderately differentiated; G3 – Poorly differentiated; cHR – Composite semi-quantitative expression of hormone receptors; *N* – Lymph node metastasis; PR – Progesterone receptor; *T* – Tumour size

**Table 6.** Univariable and multivariable time-dependent Cox analyses of factors associated with all-cause death in breast cancer patients (*n* = 572).

	Univariable		Multivariable			
Factor			Model 1^a^		Model 2^b^	
	HaR (95%CI)	*p*	HaR (95%CI)	*p*	HaR (95%CI)	*p*
Hierarchical risk stratification by cHR and histological grade		<0.0005				
Triple-negative	1					
cHR >4+ or cHR ≤4+ G1	0.325 (0.205–0.515)	<0.0005				
cHR ≤4+ G2 or G3	0.803 (0.497–1.297)	0.370				
Hierarchical risk stratification by histological grade and PR expression		<0.0005				
Triple-negative	1					
G1 or G2 PR>1+	0.305 (0.190–0.491)	<0.0005				
G2 PR ≤1+or G3	0.784 (0.493–1.248)	0.305				
Hierarchical risk stratification by histological grade and cHR		<0.0005				
Triple-negative	1					
G1 or G2 cHR>4+	0.307 (0.190–0.495)	<0.0005				
G2 cHR ≤4+ orG3	0.745 (0.470–1.181)	0.210				
Hierarchical risk stratification by Ki-67 and PR		<0.0005				<0.0005
Triple-negative	1				1	
Ki-67≤12% or Ki-67>12% PR>1+	0.341 (0.219–0.532)	<0.0005			0.246 (0.153–0.396)	<0.0005
Ki-67>12% PR≤1+	0.950 (0.571–1.580)	0.843			0.740 (0.441–1.243)	0.255
Hierarchical risk stratification by Ki-67 and cHR		<0.0005				
Triple-negative	1					
Ki-67≤12% or Ki-67>12% cHR>4+	0.331 (0.210–0.520)	<0.0005				
Ki-67>12% cHR≤4+	0.888 (0.542–1.454)	0.637				
St Gallen classification (Former)		<0.0005				
Triple-negative	1					
Ki-67≤12% and PR >1+	0.255 (0.135–0.481)	0.001				
Ki-67>12% and/or PR ≤1+	0.548 (0.359–0.836)	0.005				
St Gallen classification (modern)		<0.0005		<0.0005		
Triple-negative	1		1			
Ki-67<20% and PR >1+	0.240 (0.131–0.439)	<0.0005	0.193 (0.103–0.360)	<0.0005		
Ki-67≥20% and/or PR ≤1+	0.598 (0.390–0.916)	0.018	0.437 (0.279–0.683)	0.001		
Age						
<70 years	1		1		1	
≥70 years	1.558 (0.961–2.525)	0.072	1.689 (1.017–2.804)	0.043	1.919 (1.159–3.178)	0.011
*T*		<0.0005		0.003		0.002
*T*1	1		1		1	
*T*2–*T*3	3.332 (1.760–6.308)	<0.0005	2.210 (1.137–4.299)	0.019	2.207 (1.139–4.275)	0.019
T4	9.120 (4.429–18.781)	<0.0005	3.903 (1.772–8.596)	0.001	4.120 (1.872–9.068)	<0.0005
*N*		<0.0005		<0.0005		<0.0005
*N*0	1		1		1	
*N*1	2.354 (1.430–3.874)	0.001	1.816 (1.082–3.046)	0.024	1.872 (1.119–3.130)	0.017
*N*2	4.590 (2.669–7.897)	<0.0005	3.976 (2.228–7.096)	<0.0005	3.918 (2.212–6.940)	<0.0005
*N*3	7.175 (3.791–13.580)	<0.0005	5.039 (2.466–10.299)	<0.0005	5.501 (2.671–11.329)	<0.0005
T_COV	0.944 (0.906–0.984)	0.007	0.725 (0.303–1.734)	0.469	0.727 (0.298–1.771)	0.482

a Two-block model: first with the Stepwise Forward Wald method with entry and exit *p*-values of <0.10 and <0.05, respectively, using published factors

b Two-block model: first with the Stepwise Forward Wald method with entry and exit *p*-values of <0.10 and <0.05, respectively

Legends: G1 – Well differentiated; G2 – Moderately differentiated; G3 – Poorly differentiated; cHR – Composite semi-quantitative expression of hormone receptors; *N* – Lymph node metastasis; PR – Progesterone receptor; *T* – Tumour size

**Table 7.** Univariable and multivariable time-dependent Cox analyses of factors associated with distant metastasis in breast cancer patients in the presence of adjuvant treatments (*n* = 419).

	Univariable		Multivariable			
Factor			Model 1^a^		Model 2^b^	
	HaR (95%CI)	*p*	HaR (95%CI)	*p*	HaR (95%CI)	*p*
Stratification by cHR and histological grade		0.090				
Triple-negative	1					
cHR >4+ or cHR ≤4+ G1	0.500 (0.259–0.964)	0.039				
cHR ≤4+ G2 or G3	0.795 (0.387–1.632)	0.532				
Stratification by histological grade and PR expression		0.038				
Triple-negative	1					
G1 or G2 PR>1+	0.466 (0.240–0.905)	0.024				
G2 PR ≤1+or G3	0.874 (0.434–1.761)	0.706				
Stratification by histological grade and cHR		0.045				
Triple-negative	1					
G1 or G2 cHR>4+	0.465 (0.238–0.909)	0.025				
G2 cHR ≤4+ or G3	0.841 (0.421–1.682)	0.625				
Stratification by Ki-67 and PR		0.002		<0.0005		
Triple-negative	1		1			
Ki-67≤12% or Ki-67>12% PR>1+	0.436 (0.227–0.839)	0.013	0.234 (0.112–0.489)	<0.0005		
Ki-67>12% PR≤1+	1.242 (0.605–2.549)	0.555	0.711 (0.318–1.593)	0.408		
Stratification by Ki-67 and cHR		0.004				
Triple-negative	1					
Ki-67≤12% or Ki-67>12% cHR>4+	0.427 (0.220–0.830)	0.012				
Ki-67>12% cHR≤4+	1.105 (0.549–2.224)	0.781				
Stratification by grade, Ki-67 and PR		0.003				
Triple-negative	1					
G1/G2 Ki-67≤12% or G1/G2 Ki-67>12%/PR>1+	0.418 (0.214–0.815)	0.011				
G1/G2 Ki-67>12%/PR≤1+ or G3	1.108 (0.554–2.215)	0.773				
Stratification by grade, Ki-67 and cHR		0.001				0.001
Triple-negative	1				1	
G1/G2 Ki-67≤12% or G1/G2 Ki-67>12%/cHR>4+	0.394 (0.200–0.776)	0.007			0.240 (0.114–0.506)	<0.0005
G1/G2 Ki-67>12%/cHR≤4+ or G3	1.118 (0.567–2.204)	0.748			0.520 (0.238–1.132)	0.099
St Gallen classification (Former)		0.176				
Triple-negative	1					
Ki-67≤12% and PR >1+	0.486 (0.222–1.061)	0.070				
Ki-67>12% and/or PR ≤1+	0.634 (0.335–1.201)	0.162				
St Gallen classification (modern)		0.150				
Triple-negative	1					
Ki-67<20% and PR >1+	0.475 (0.224–1.008)	0.053				
Ki-67≥20% and/or PR ≤1+	0.656 (0.345–1.250)	0.200				
Age						
≤40 years	1		1		1	
>40 years	0.385 (0.186–0.794)	0.010	0.639 (0.289–1.410)	0.267	0.685 (0.312–1.502)	0.345
*T*		<0.0005		0.021		0.042
*T*1	1		1		1	
*T*2–*T*3	3.650 (1.775–7.506)	<0.0005	2.675 (1.188–6.023)	0.017	2.499 (1.122–5.566)	0.025
*T*4	12.876 (4.296–38.591)	<0.0005	5.911 (1.503–23.252)	0.011	4.509 (1.179–17.245)	0.028
*N*		<0.0005		<0.0005		<0.0005
*N*0	1		1		1	
*N*1	3.765 (1.845–7.680)	<0.0005	2.896 (1.359–6.170)	0.006	2.961 (1.394–6.290)	0.005
*N*2/*N*3	11.772 (5.842–23.719)	<0.0005	10.877 (4.836–24.465)	<0.0005	11.182 (4.948–25.721)	<0.0005
Systemic treatment						
Inadequate	1		1		1	
Adequate	0.345 (0.169–0.707)	0.004	0.715 (0.310–1.649)	0.432	0.942 (0.389–2.280)	0.894
Local radiotherapy						
Inadequate	1		1		1	
Adequate	0.625 (0.267–1.465)	0.279	0.224 (0.083–0.604)	0.003	0.221 (0.083–0.585)	0.002
T_COV	0.937 (0.877–1.001)	0.054	0.413 (0.023–7.354)	0.547	0.418 (0.027–6.574)	0.535

a Two-block model: first with the Stepwise Forward Wald method with entry and exit *p*-values of <0.10 and <0.05, respectively; second block with the insert method

b Similar to the first model, but excluding the risk classification obtained in the last model

Legends: G1 – Well differentiated; G2 – Moderately differentiated; G3 – Poorly differentiated; cHR – Composite semi-quantitative expression of hormone receptors; *N* – Lymph node metastasis; PR – Progesterone receptor; *T* – Tumour size

**Table 8.** Univariable and multivariable time-dependent Cox analyses of factors associated with all-cause death in breast cancer patients in the presence of adjuvant treatments (*n* = 419).

	Univariable		Multivariable			
Factor			Model 1^a^		Model 2^b^	
	HaR (95%CI)	*p*	HaR (95%CI)	*p*	HaR (95%CI)	*p*
Stratification by cHR and histological grade		0.015				
Triple-negative	1					
cHR >4+ or cHR ≤4+ G1	0.479 (0.249–0.924)	0.028				
cHR ≤4+ G2 or G3	1.061 (0.539–2.087)	0.864				
Stratification by histological grade and PR expression		0.019				
Triple-negative	1					
G1 or G2 PR>1+	0.484 (0.251–0.933)	0.030				
G2 PR ≤1+or G3	1.043 (0.530–2.055)	0.902				
Stratification by histological grade and cHR		0.012				
Triple-negative	1					
G1 or G2 cHR>4+	0.462 (0.237–0.900)	0.023				
G2 cHR ≤4+ or G3	1.044 (0.537–2.031)	0.899				
Stratification by Ki-67 and PR		0.006				
Triple-negative	1					
Ki-67≤12% or Ki-67>12% PR>1+	0.494 (0.261–0.936)	0.030				
Ki-67>12% PR≤1+	1.270 (0.624–2.586)	0.510				
Stratification by Ki-67 and cHR		0.001		0.002		
Triple-negative	1		1			
Ki-67≤12% or Ki-67>12% cHR>4+	0.445 (0.231–0.858)	0.016	0.308 (0.150–0.635)	0.001		
Ki-67>12% cHR≤4+	1.302 (0.662–2.561)	0.445	0.770 (0.373–1.588)	0.479		
Stratification by grade, Ki-67 and PR		0.006				
Triple-negative	1					
G1/G2 Ki-67≤12% or G1/G2 Ki- 67>12%/PR>1+	0.475 (0.248–0.911)	0.025				
G1/G2 Ki- 67>12%/PR≤1+ or G3	1.170 (0.590–2.322)	0.652				
Stratification by grade, Ki-67 and cHR		0.001				0.001
Triple-negative	1				1	
G1/G2 Ki-67≤12% or G1/G2 Ki-67>12%/cHR>4+	0.429 (0.221–0.835)	0.013			0.291 (0.139–0.608)	0.001
G1/G2 Ki-67>12%/cHR≤4+ or G3	1.251 (0.643–2.433)	0.510			0.761 (0.374–1.550)	0.452
St Gallen classification (Former)		0.131				
Triple-negative	1					
Ki-67≤12% and PR >1+	0.447 (0.202–0.985)	0.046				
Ki-67>12% and/or PR ≤1+	0.754 (0.404–1.409)	0.376				
St Gallen classification (modern)		0.060				
Triple-negative	1					
Ki-67<20% and PR >1+	0.411 (0.190–0.891)	0.024				
Ki-67≥20% and/or PR ≤1+	0.811 (0.433–1.581)	0.512				
Age						
<70 years	1		1		1	
≥70 years	1.856 (1.022–3.370)	0.042	1.914 (1.007–3.639)	0.048	1.924 (1.013–3.654)	0.045
*T*		<0.0005		0.029		0.024
*T*1	1		1		1	
*T*2–*T*3	3.108 (1.555–6.213)	0.001	2.357 (1.113–4.990)	0.025	2.412 (1.141–5.100)	0.021
*T*4	13.088 (4.417–38.782)	<0.0005	4.867 (1.323–17.908)	0.017	5.129 (1.387–18.970)	0.014
*N*		<0.0005		0.001		0.001
*N*0	1		1		1	
*N*1	2.356 (1.262–4.398)	0.007	1.887 (0.983–3.623)	0.056	1.833 (0.955–3.519)	0.068
*N*2/*N*3	5.840 (3.083–11.062)	<0.0005	4.381 (2.074–9.257)	<0.0005	5.129 (1.387–18.970)	<0.0005
Systemic treatment						
Inadequate	1		1		1	
Adequate	0.252 (0.130–0.487)	<0.0005	0.818 (0.365–1.836)	0.626	0.816 (0.364–1.832)	0.623
Local radiotherapy						
Inadequate	1		1		1	
Adequate	0.524 (0.224–1.224)	0.135	0.317 (0.126–0.801)	0.015	0.309 (0.122–0.782)	0.013
T_COV	0.936 (0.879–0.996)	0.036	0.656 (0.206–2.091)	0.476	0.656 (0.204–2.105)	0.479

a Two-block model: first with the Stepwise Forward Wald method with entry and exit *p*-values of <0.10 and <0.05, respectively; second block with the insert method

b Similar to the first model, but excluding the risk classification obtained in the last model

Legends: G1 – Well differentiated; G2 – Moderately differentiated; G3 – Poorly differentiated; cHR – Composite semi-quantitative expression of hormone receptors; *N* – Lymph node metastasis; PR – Progesterone receptor; *T* – Tumour size
